# Persistent Vascular Complications in Long COVID: The Role of ACE2 Deactivation, Microclots, and Uniform Fibrosis

**DOI:** 10.3390/idr16040042

**Published:** 2024-06-27

**Authors:** Christina-Michailia Sideratou, Christos Papaneophytou

**Affiliations:** Department of Life Sciences, School of Life and Health Sciences, University of Nicosia, 2417 Nicosia, Cyprus; christin.sideratou@gmail.com

**Keywords:** long COVID, severe acute respiratory syndrome-coronavirus-2, ACE2, RAS, vasoregulation, microclots, fibrosis

## Abstract

Angiotensin-converting enzyme 2 (ACE2), a key regulator in vasoregulation and the renin–angiotensin system, is hypothesized to be downregulated in patients with COVID-19, leading to a cascade of cardiovascular complications. This deactivation potentially results in increased blood pressure and vessel injury, contributing to the formation and persistence of microclots in the circulation. Herein, we propose a hypothesis regarding the prolonged vascular complications observed in long COVID, focusing on the role of ACE2 deactivation and/or shedding, the persistence of microclots, and the unique pattern of fibrosis induced by severe acute respiratory syndrome-coronavirus-2 (SARS-CoV-2). Furthermore, we propose that the distinctive, uniform fibrosis associated with COVID-19, which is challenging to detect through conventional X-ray imaging, exacerbates vascular injury and impairs oxygenation. The persistence of these microclots and the unique fibrosis pattern are suggested as key factors in the extended duration of vascular complications post-COVID-19 infection, regardless of the initial disease severity. Moreover, plasma ACE2 activity has the potential to serve as prognostic or diagnostic biomarkers for monitoring disease severity and managing long COVID symptoms. Elucidating the role of ACE2 deactivation and the consequent events is vital for understanding the long-term effects of COVID-19. The experimental verification of this hypothesis through in vitro studies, clinical longitudinal studies, and advanced imaging techniques could yield significant insights into the pathophysiological mechanisms underlying long COVID, thereby improving the management of patients, particularly those with cardiovascular complications.

## 1. Introduction

In December 2019, a novel coronavirus known as severe acute respiratory syndrome-coronavirus-2 (SARS-CoV-2) initiated an outbreak of pneumonia in Wuhan, China [1]. This virus caused coronavirus disease 2019 (COVID-19), rapidly transforming from an outbreak into a global pandemic with considerable mortality and morbidity [2]. The clinical characteristics of COVID-19 range from asymptomatic cases to critical illness; about 80% of affected individuals experience mild to moderate disease, including fever, cough, sore throat, headache, and nasal congestion [3]. On the other hand, approximately 5% of infected individuals develop critical illness, including severe cases involving pneumonia, respiratory failure requiring mechanical ventilation, multi-organ failure, kidney damage, and death [3].

Typically, the initial active stage of COVID-19 extends for about four weeks after the onset of the infection [4]. Some individuals report symptoms that persist beyond the initial infection, entering the ‘long COVID’ phase. Notably, these extended symptoms can last several weeks to months after the first infection, regardless of its initial severity [5].

We recently reviewed the persisting symptoms of COVID-19, characterizing long COVID as a multi-organ disruptor that affects the respiratory, cardiovascular, and central nervous systems [6]. While the exact mechanisms leading to long COVID remain inconclusive, several studies have highlighted the role of angiotensin-converting enzyme 2 (ACE2), the entry point of the virus into host cells, in the development of the disease [7]. Herein, we present a hypothesis explaining how the dysregulation of ACE2 is associated with persistent vascular complications in long COVID.

## 2. Vascular Complications in Long COVID

It has been demonstrated that SARS-CoV-2 triggers the activation of coagulation through procoagulant factors and proinflammatory cytokines, increasing the risk of thrombosis, ischemia, and atherosclerotic plaque rupture [8]. Elevated levels of proinflammatory cytokines, such as interleukins (IL-2, IL-7, and others), have been detected in patients with COVID-19 in intensive care units (ICUs) [9]. Additionally, those with severe disease manifestations, especially those experiencing sepsis-induced coagulopathy and disseminated intravascular coagulation (DIC), are more likely to develop coagulation abnormalities due to the virus’s inflammatory response [10]. This inflammation-driven response can enhance coagulation cascades, exacerbating the risk of thrombosis and associated complications.

Notably, coagulopathies, particularly characterized by the extensive formation of microclots in vivo, are a key feature of both COVID-19 and long COVID, and studies have shown that these microclots possess amyloid-like properties [11]. Importantly, introducing purified, recombinant SARS-CoV-2 S1 spike protein into normal plasma capable of clotting can trigger the formation of abnormal clots. These clots not only assume amyloid states but also show resistance to fibrinolysis, the process that breaks down blood clots [12]. Table 1 summarizes the results from some studies investigating the prolonged impact of COVID-19 on thrombotic incidents and coagulation markers, highlighting increased risks and abnormalities that persist well beyond the initial infection period.

Recent studies have demonstrated an elevation in various markers showing thrombotic incidents within the microcirculation that continue for several months following the acute phase of the infection with SARS-CoV-2 [13,19,20]. Korompoki et al. [21] summarized the evidence of post-acute hematological complications of COVID-19 and confirmed that persistent coagulation abnormalities and thrombosis are common in long COVID. In the cohort study by Townsend et al. [14], 150 patients were assessed over an average of 80.5 days (ranging from 44 to 155 days) following their initial diagnosis. It was observed that 23.3% of the patients had increased D-dimer levels (>500 ng/mL) up to four months after being infected with SARS-CoV-2.

The increase in D-dimer levels was also confirmed by the prospective cohort study conducted by Pasini et al. [15] involving 75 patients who had been hospitalized with laboratory-confirmed mild to moderate COVID-19 and were examined 60 days later. All patients (100%) exhibited very high serum ferritin and D-dimer concentrations. The study further found that 87% of the patients had clinically significant low levels of hemoglobin and 72% had low levels of albumin. Additionally, 73% of the patients showed elevated rates of erythrocyte sedimentation and C-reactive protein (CRP).

In the prospective observational study by Fan et al. [16], the hemostatic, endothelial, and inflammatory profiles of 39 patients who had recovered from COVID-19 were evaluated up to 16 months post-recovery and compared with a control group of 124 healthy volunteers. The study found significantly elevated biomarkers of hypercoagulability, endothelial disease, and inflammation in the recovered patients. Specifically, 17.9% (7/39) and 48.7% (19/39) of the patients had higher levels of the D-dimer and antihemophilic factor (VIII), respectively, above normal limits. Additionally, the median endogenous thrombin potential (ETP) was higher, and antithrombin levels were significantly lower (97%; *p* < 0.001) compared to the control group. The study of von Meijenfeldt et al. [22] revealed increased thrombin production and decreased plasma fibrinolytic potential four months post-discharge, indicating prolonged prothrombotic changes. Elevated levels of factor VIII and Plasminogen Activator Inhibitor type-1 (PAI-1) were also observed, suggesting sustained endothelial cell activation, contributing to the hypercoagulability and hypofibrinolytic state [17]. In addition to microvascular dysfunction, macrovascular episodes appear to be a significant contributor to long COVID symptoms.

Xie et al. [23] evaluated the cardiovascular risks and burdens among 153,760 individuals with COVID-19 using national healthcare databases from the US Department of Veterans Affairs, comparing them with two control cohorts totaling over 11 million individuals. They found that beyond the first 30 days post-infection, individuals with COVID-19 showed an increased risk of various cardiovascular diseases, including cerebrovascular disorders, dysrhythmias, both ischemic and non-ischemic heart disease, pericarditis, myocarditis, heart failure, and thromboembolic disease. These risks were apparent even in those who were not hospitalized during the infection and escalated according to the level of care received during the acute phase—ranging from non-hospitalized, to hospitalized, to ICU admission. Their findings highlight a substantial one-year burden of cardiovascular conditions in COVID-19 survivors, suggesting that post-recovery care should emphasize cardiovascular health.

Katsoularis et al. [24] also highlighted an increased risk of venous thrombosis following COVID-19 infection, with a significant spike in the incidence of deep vein thrombosis, pulmonary embolism, and bleeding, especially within the first 30 days post-infection. The incidence rate ratios were notably high, reaching up to 46.40 for a pulmonary embolism in the second week. The adjusted risk ratios for these conditions also showed elevated levels, with a pulmonary embolism presenting an adjusted ratio of 33.05. These risks were higher among patients with critical COVID-19 and during the first wave of the pandemic in Sweden. The absolute risks were 0.039% for deep vein thrombosis, 0.17% for a pulmonary embolism, and 0.101% for bleeding. These findings underscore COVID-19 as a significant risk factor for a venous thromboembolism and underscore the need for enhanced diagnostic and prophylactic strategies to manage post-infection complications.

It is worthwhile to mention that despite that COVID-19 vaccines have been associated with rare vascular complications, such as vaccine-induced immune thrombotic thrombocytopenia (VITT), the incidence of these events is exceedingly low compared to the risk of vascular complications from COVID-19 itself [25]. VITT involves abnormal clotting and low platelet counts, with an incidence estimated at 1 in 100,000 to 1 in 1,000,000 doses for adenovirus vector-based vaccines [26]. Thrombosis and vasculitis have also been reported post-vaccination, but these events are rare and often occur in individuals with pre-existing risk factors. The risk of severe vascular events, such as deep vein thrombosis (DVT), pulmonary embolism (PE), and stroke, is significantly higher in patients with COVID-19 due to the virus-induced endothelial dysfunction and hypercoagulability [27]. The benefits of vaccination in preventing severe disease, hospitalization, and death far outweigh the risks of these rare adverse events [28].

## 3. The Role of ACE2 in COVID-19 and Long COVID

**Figure 1:**
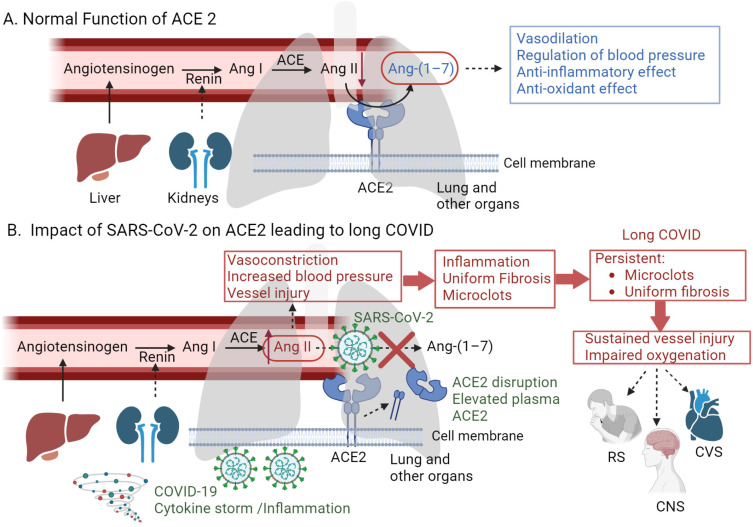
A graphical representation of the proposed hypothesis. (**A**) Normal function of ACE2 in the renin–angiotensin system (RAS): Renin converts angiotensinogen to angiotensin I (Ang I). Ang I is then converted to angiotensin II (Ang II) by ACE, which is present on the surfaces of endothelial cells, primarily in the lungs and kidneys. ACE2 acts as a counter-regulator by converting Ang II to angiotensin 1–7 (Ang-(1–7)), which exhibits vasodilatory and anti-inflammatory activities, thus regulating blood pressure. (**B**) Impact of SARS-CoV-2 on ACE2 leading to long COVID: SARS-CoV-2 binds to ACE2, reducing its presence on the endothelium of the lung and other organs, which disrupts the normal function of ACE2. This increases Ang II levels, leading to vasoconstriction, vessel injury, and inflammation. Moreover, the binding of SARS-CoV-2 to ACE2 causes the shedding of these receptors by various proteases, leading to elevated levels of plasma ACE2. These events contribute to the formation of persistent microclots and uniform fibrosis, leading to impaired oxygenation—a hallmark symptom of long COVID that affects various systems, including the respiratory (RS), central nervous (CNS), and cardiovascular (CVS) systems (created with BioRender.com, accessed on 1 April 2024).

ACE2 is essential in the renin–angiotensin system (RAS), influencing physiological and pathological processes in the heart, kidneys, lungs, and other organs [29]. As a critical counter-regulatory enzyme, ACE2 mitigates the actions of ACE by converting angiotensin II (Ang II)—a molecule that constricts blood vessels and increases blood pressure—into angiotensin 1–7 (Ang-(1–7)), which has the opposite effect (Figure 1A). Ang-(1–7) promotes vasodilation, helping to maintain blood pressure homeostasis and offering protection against vascular inflammation and thrombosis [30]. Additionally, the enzyme’s activity leads to the stimulation of the Mas receptor, which contributes to lowered blood pressure and lessened inflammation and fibrosis, highlighting its crucial role in vascular and systemic homeostasis [31,32].

Moreover, ACE2 serves as a protective mechanism against tissue damage, and its reduced expression is linked to various health issues, including pulmonary edema, acute respiratory distress syndrome, atherosclerosis, hypertension, cardiac hypertrophy, ventricular remodeling, and heart failure, as reported in several studies [33,34]. Additionally, mice genetically engineered to lack ACE2 showed a heightened vulnerability to cardiac damage and more severe results in different models of inflammation and injury, such as influenza-induced lung damage and acute respiratory distress syndrome [35]. When subjected to an acid challenge, these ACE2-deficient mice demonstrated significantly increased lung stiffness, deteriorated oxygen levels, severe lung edema, and heightened inflammatory responses [36]. Similarly, mice infected with the SARS-CoV virus, or its S protein, exhibited severe symptoms like those of ACE2-deficient mice, including worse outcomes from acid-induced lung damage characterized by structural changes in the lung tissue, increased lung edema, and enhanced leukocyte presence [37]. Notably, ACE2 levels were substantially decreased in the lungs of these SARS-CoV-infected mice, suggesting that the down-regulation of ACE2 may play a critical role in the severe pathological effects seen with SARS-CoV infections [37].

The significance of ACE2 extends into the pathogenesis of COVID-19, particularly in understanding the prolonged effects of long COVID. SARS-CoV-2 engages the ACE2 receptor for entry, leading to direct cellular damage from the virus and triggering the release of an excessive number of immune mediators such as interleukins (e.g., IL-1β, IL-2R, IL-6, IL-10) [38], tumor necrosis factor-alpha (TNF-α) [39], and interferons (e.g., IFN-γ) [40], along with abnormalities in blood clotting [41]. The presence of the virus within lung cells corroborates this destructive process [42]. The subsequent release of immune molecules [43] is believed to initiate a cytokine storm, characterized by heightened levels of interleukins, tumor necrosis factor, and interferons [39]. A commonly seen consequence in patients is immune-driven widespread blood clotting within vessels, resulting in lung micro-thrombosis [44]. It has been suggested that the binding of the coronavirus spike protein to ACE2 leads to the shedding of ACE2 receptors by various proteases, which leads to the loss of protective function of the ACE2/MAS axis in the lungs and other organs [45]. Furthermore, since SARS-CoV-2 utilizes ACE2 receptors for cell entry, this interaction reduces ACE2 levels on cell surfaces [46]. This downregulation disrupts vasoregulation, potentially resulting in heightened vascular inflammation and coagulation abnormalities [46,47]. Such disturbances can contribute to the cardiovascular and other complications often seen in long COVID including persistent hypertension and increased thrombotic risk [48]. Given the pivotal role of ACE2 in both vasoregulation and COVID-19 pathogenesis, understanding its functions and interactions is essential for developing targeted therapies to mitigate long COVID symptoms, particularly those related to cardiovascular health. Therapeutic approaches that aim to restore the balance of RAS or compensate for the loss of ACE2 activity might benefit patients with long COVID [49].

Once the virus enters the host cell, it reduces ACE2 expression, leading to an increase in Ang II, which in turns binds to its receptor, Ang II receptor type 1, and influences the expression of various matrix metalloproteinases like MMP-1 and MMP-3, and inflammatory cytokines such as TNF-α, IL-6, IL-8, IL-1, and MCP1 through nuclear factor κB signaling [50,51]. Importantly, this inflammatory stimulus can disrupt the antithrombotic equilibrium, fostering inflammatory environments that may culminate in thrombus formation [51,52]. Factors such as cytokine signaling, hypoxia, tissue damage, and damage-associated molecular patterns (DAMPs) can cause endothelial cells (ECs) to switch from an anticoagulant to a procoagulant state [53]. During inflammation, several processes including the loss of vascular integrity, diminished expression of antithrombotic agents, interactions between neutrophils and platelets, platelet activation, tissue factor (TF) expression in monocytes, release of microparticles, and inhibition of fibrinolysis contribute to immunothrombosis [54].

It is worthwhile to mention that current COVID-19 treatments may affect ACE2 expression and function in various ways. Antiviral medications like Remdesivir reduce viral load without directly impacting ACE2 levels, potentially preventing the further downregulation of ACE2 [55]. It has been demonstrated that corticosteroids including dexamethasone may upregulate ACE2 both in vitro and in vivo [56]. Monoclonal antibodies like tocilizumab, targeting IL-6 receptors, also reduce inflammation; however, these may have a minor impact on ACE2 concentrations [57]. RAS modulators, such as ACE inhibitors and Ang II receptor blockers, might upregulate ACE2 expression, potentially offering protective effects despite theoretical concerns about enhanced viral entry [58]. Other therapeutics, such as convalescent plasma, focus on enhancing immune response without a well-defined impact on ACE2 [59]. Understanding these interactions is crucial for optimizing COVID-19 treatment strategies to preserve ACE2 function and mitigate long-term vascular complications associated with long COVID.

## 4. Hypothesis: ACE2 Deactivation or Shedding Leads to Cardiovascular Complications

Herein, we hypothesize that in individuals recovering from COVID-19, the deactivation/shedding of ACE2 plays a pivotal role in prolonged cardiovascular and other complications characteristic of long COVID (Figure 1B). Significantly, clinical observations have identified various factors, such as older age, hypertension, diabetes, and cardiovascular disease, which are linked to both the severity and progression of COVID-19 and also correlate with differing levels of ACE2 deficiency [60]. ACE2 receptors in kidneys may also explain significant renal dysfunction in severely affected patients [61]. Furthermore, ACE2, which is located on vascular endothelial cells, undergoes proteolytic shedding, resulting in a soluble form present in the plasma, known as soluble ACE2 or serum/plasma ACE2. Plasma ACE2 is the ectodomain—the part of the enzyme that extends outside the cell—that has been cleaved from the cell surface through shedding [33]. While the precise reasons for this shedding are not fully understood [62], it is observed to occur more frequently in conditions such as hypertension and heart disease [63]. Studies of plasma ACE2 levels in healthy individuals are limited, but most findings indicate that levels range from undetectable to very low [64]. In contrast, patients with cardiovascular risk factors or diseases exhibit increased circulating ACE2, which is linked to adverse outcomes. Interestingly, it has been reported that patients with COVID-19 show elevated circulating ACE2 levels compared to non-COVID-19 controls, which persist for at least eight months [65]. Given that COVID-19 is associated with a hypercoagulable state, this suggests the possibility of a relationship between plasma ACE2 activity and coagulation profiles. We, therefore, hypothesize the following sequence of events following an infection with SARS-CoV-2 leading to long COVID:

- ACE2, vital for vasoregulation, is deactivated during COVID-19 [66], leading to sustained blood pressure elevation and the formation of microclots. The deactivation results from the virus binding to ACE2 receptors, leading to their downregulation and a loss of catalytic function at the cell surface [29]. This disruption impacts the counter-regulatory pathway of RAS, which is critical in controlling blood pressure, inflammation, and fibrosis, and contributes to the pathology of hypertension, cardiovascular disease, and chronic kidney disease [67].
- The microclots, composed of fibrin and other materials generated by vessel injury, persist for months following the subsidence of lung inflammation, indicating ongoing vascular injury. This could be partly attributed to oxygen deprivation due to fibrosis [68].
- These persistent symptoms are more likely to result in post-COVID-19 pulmonary fibrosis (PCPF). Interestingly, PCPF has been reported in approximately 44.9% of COVID-19 survivors. Patients with fibrosis frequently experience persistent symptoms such as dyspnea, cough, chest pain, fatigue, and myalgia [69]. Notably, the fibrosis pattern in COVID-19 differs markedly from that seen in occupational settings [70]. It is more uniform and less detectable by conventional imaging (i.e., X-ray), and indicates a distinct pathological mechanism. This uniform fibrosis could further exacerbate the lack of oxygen and contribute to vascular complications [71].
- The entry of SARS-CoV-2 into cells through membrane fusion reduces ACE2 receptors, leading to increased pulmonary inflammation and coagulation, as evidenced by heightened Ang II activity [41]. This is supported by the discovery of viral particles in lung tissue [72] and the immune response, including a cytokine storm [43] with elevated interleukins, tumor necrosis factor, and interferons, contributing to immune-mediated coagulation disturbances such as disseminated intravascular coagulation with lung micro-thrombosis [67].

This hypothesis underscores the vital role of ACE2 in the development and persistence of vascular complications in long COVID, highlighting the need for targeted therapeutic strategies.

## 5. Implementations and Suggestions

We propose several specific approaches to verify and validate the hypothesis presented. These strategies aim to investigate further and manage the role of ACE2 deactivation, microclots, and uniform fibrosis in long COVID:

- Conduct in vitro studies using cell cultures to directly observe the impact of SARS-CoV-2 on ACE2 receptor expression and function. Techniques such as CRISPR-Cas9 can manipulate ACE2 expression in endothelial cells, followed by viral infection assays to monitor changes.
- Implement longitudinal studies tracking patients with COVID-19 over time. Parameters to assess include blood pressure, microclot formation, and fibrosis progression. Specific biomarkers to measure include ACE2 activity and concentration in blood circulation, D-dimer levels to monitor coagulation status, and the levels of specific inflammatory cytokines such as TNF-α, IL-6, IL-8, IL-1, and MCP1, and interferons such as IFN-γ.
- Combine imaging biomarkers with advanced imaging modalities, such as high-resolution computed tomography (HRCT) and magnetic resonance imaging (MRI), to detect subtle changes in fibrosis. Use inflammatory biomarkers alongside imaging for a comprehensive assessment.
- Explore the potential utility of RAS modulators, such as ACE inhibitors and angiotensin II receptor blockers (ARBs), in counteracting ACE2 deactivation.
- Evaluate the efficacy of anticoagulant therapy for managing microclot-related complications, particularly in high-risk patients. Assess the role of anti-inflammatory drugs in mitigating cytokine storms and reducing vascular inflammation.
- Implement long-term monitoring of COVID-19 survivors to enable the early detection and management of potential vascular complications. Personalized medicine strategies should consider individual risk factors, such as age, existing comorbidities, and the severity of the initial infection.
- Leverage emerging technologies like artificial intelligence (AI) and machine learning (ML) for predictive modeling and developing personalized treatment strategies. AI and ML can analyze large datasets to identify patterns and predict outcomes, enhancing the management of long COVID.

Nevertheless, collaborative research projects and international databases for sharing patient data and research findings are essential.

## 6. Conclusions

The hypothesis presented herein posits a central role for ACE2 in the pathophysiology of long COVID. This hypothesis could partially explain the mechanisms leading to persistent symptoms following a SARS-CoV-2 infection if verified. ACE2 is crucial for vasoregulation, and its deactivation can lead to increased blood pressure and vessel injury, resulting in the formation of microclots. In cases of long COVID, these microclots could persist in the bloodstream months after the initial lung inflammation has subsided, suggesting ongoing vascular injury. This may be attributed to reduced oxygenation due to fibrosis, often non-uniform and not easily detected through X-ray examination. Experimentally pursuing this hypothesis, as proposed here, or through other experimental approaches, could illuminate the role of ACE2 in developing long COVID. Furthermore, plasma ACE2 activity could serve as a prognostic or diagnostic biomarker to monitor the severity of the disease or to aid in managing and diagnosing long COVID symptoms. Moreover, it is essential to acknowledge that SARS-CoV-2 continues to circulate, and patients may still develop long COVID complications even after recent mild infections.

## Figures and Tables

**Table 1:** Examples of studies investigating the persistent thrombotic and coagulation abnormalities in post-COVID-19 patients.

Study	Study Characteristic	Key Findings	Ref
1	Blood samples of 46 COVID-19 pneumonia hospitalization survivors were collected 77.5 days after symptom onset	Persistent microclot formation was a hallmark in patients with COVID-19 and long COVID	[13]
2	150 patients were assessed 44–155 days post-diagnosis	Elevated D-dimer levels in 25.3% of patients up to 4 months post-infection Higher D-dimer levels were associated with patients > 50 years of age and those who had required hospitalization	[14]
3	Two months post-hospital discharge, 75 previously confirmed patients with COVID-19 reported new symptoms of fatigue, muscle weakness, and/or dyspnea	All showed high levels of serum ferritin and D-dimer; 87% had low hemoglobin and 72% had low albumin levels	[15]
4	Hemostatic, endothelial, and inflammatory profiles of 39 recovered patients, evaluated up to 16 months post-recovery	Significantly higher levels of D-dimer and factor VIII; increased thrombin production capacity and reduced plasma fibrinolytic potential	[16]
5	Study on coagulation markers 4 months after discharge	Increased thrombin production capacity and sustained activation of endothelial cells	[17]
6	Large-scale statistical analysis over 90 days post-infection (n = 4906)	Thromboembolic events and mortality were common post-discharge in inpatients with COVID-19, with a 7.13% rate of combined VTE ^1^, ATE ^2^, and ACM ^3^ Risk factors included advanced age, ICU ^4^ stays, and chronic conditions Post-discharge anticoagulation, used in 13.2% of patients, reduced thromboembolic events and mortality by 46%	[18]

^1^ VTE: Venous thromboembolism; ^2^ ATE: Arterial thromboembolism; ^3^ ACM: All-cause mortality; ^4^ ICU: Intensive care unit.

## Data Availability

No new data were created in this study.
